# A Review: Genetic Mutations as a Key to Unlocking Drug Resistance in Cervical Cancer

**DOI:** 10.1177/10732748241261539

**Published:** 2024-06-16

**Authors:** Carla Eksteen, Johann Riedemann, Atarah M Rass, Manisha du Plessis, Matthys H Botha, Frederick H van der Merwe, Anna-Mart Engelbrecht

**Affiliations:** 1 CancerCare, Cape Gate Oncology Centre, Cape Town, South Africa; 2 Department of Physiological Sciences, Faculty of Science, 98826 University of Stellenbosch, Stellenbosch, South Africa; 3 Department of Obstetrics and Gynecology, 26697 Stellenbosch University, Stellenbosch, South Africa; 4 African Cancer Institute (ACI), Department of Global Health, Faculty of Medicine and Health Sciences, 26697 Stellenbosch University, Stellenbosch, South Africa

**Keywords:** cervical cancer, treatment resistance, cisplatin, precision oncology, genetic mutations

## Abstract

Cervical cancer is the fourth most common cancer in women. Advanced stage and metastatic disease are often associated with poor clinical outcomes. This substantiates the absolute necessity for high-throughput diagnostic and treatment platforms that are patient and tumour specific. Cervical cancer treatment constitutes multimodal intervention. Systemic treatments such as chemotherapy and/or focal radiotherapy are typically applied as neoadjuvant and/or adjuvant strategies. Cisplatin constitutes an integral part of standard cervical cancer treatment approaches. However, despite initial patient response, *de novo* or delayed/acquired treatment resistance is often reported, and toxicity is of concern. Chemotherapy resistance is associated with major alterations in genomic, metabolomic, epigenetic and proteomic landscapes. This results in imbalanced homeostasis associated with pro-oncogenic and proliferative survival, anti-apoptotic benefits, and enhanced DNA damage repair processes. Although significant developments in cancer diagnoses and treatment have been made over the last two decades, drug resistance remains a major obstacle to overcome.

## Plain Language Summary

Despite advances in treatment, the disease’s advanced stages and spread to other parts of the body often lead to poor outcomes. This highlights the urgent need for better diagnostic and treatment methods tailored to each patient and their specific tumour. Treatment for cervical cancer usually involves a combination of therapies. Chemotherapy and focused radiation therapy are commonly used before or after surgery to improve outcomes. However, some patients develop resistance to these treatments, either from the start or after initially responding to therapy. This resistance can make treatment less effective and increase the risk of side effects. Chemotherapy resistance is often linked to changes in the genes and proteins of cancer cells. These changes disrupt the normal balance within the cells, making them more prone to grow and survive, resist cell death, and repair DNA damage caused by treatment. Despite progress in cancer research and treatment, drug resistance remains a significant challenge. This review aims to explore how acquired genetic mutations contribute to drug resistance in cervical cancer. By understanding these mutations better, researchers and clinicians in low- to middle-income countries can develop more effective treatment strategies to improve outcomes for patients.

## Introduction

Cervical cancer ranks as the fourth most prevalent cancer in women worldwide.^1^ In 2018, approximately 570,000 women were diagnosed with cervical cancer globally, with an estimated 311,000 women succumbing to the disease. Although a rise in these figures is anticipated, the bulk of the increase is foreseen to occur in low- and middle-income countries (LMICs). The primary etiological factor of cervical cancer is high-risk Human Papillomavirus (HPV) infection, particularly strains HPV-16 and 18, which are responsible for 99% of cervical cancer cases.^2,3^ Although vaccination (Cervarix/Guardisil) against HPV is poised to reduce the number of new cases, its administration remains infrequent in LMICs, particularly in sub-Saharan Africa.^4,5^

The treatment approach for cervical cancer is contingent upon the disease’s stage.^6^ Despite advancements in treatment protocols, patients with advanced-stage disease have a poor prognosis, with a 1-year survival rate ranging from 10 to 20%. Recurrence is observed in approximately 10 to 20% of patients with early-stage cervical cancer following surgery or radiotherapy, and in up to 70% of those with locally advanced-stage disease.^7^ The recurrence of the disease is primarily attributed to the presence of therapy-resistant tumour cells. As such, a comprehensive understanding of the mechanism underlying treatment resistance and the application of personalized therapy could enhance the clinical outcomes for these patients.

## Current Treatment Approaches: Cisplatin Resistance

Cervical cancer treatment constitutes multimodal intervention. Chemotherapy, especially platinum ligated drugs such as cisplatin, is an integral part of standard cervical cancer treatment of more advanced stages. Cisplatin has been used to treat cervical cancer since 1980^8^ and remains to be one of the most effective anti-cancer agents for advanced and recurrent cervical cancer. Based on tumor response to the initial therapy, cancer resistance is broadly classified into two categories, *de novo*/primary or acquired resistance.^9-11^ *De novo*/primary drug resistance exists prior to any given treatment, where acquired resistance occurs after initial therapy. Unfortunately, most patients will likely develop resistance at a certain point of treatment. Despite initial patient response to cisplatin, increased treatment resistance is often reported.

**Figure 1.**
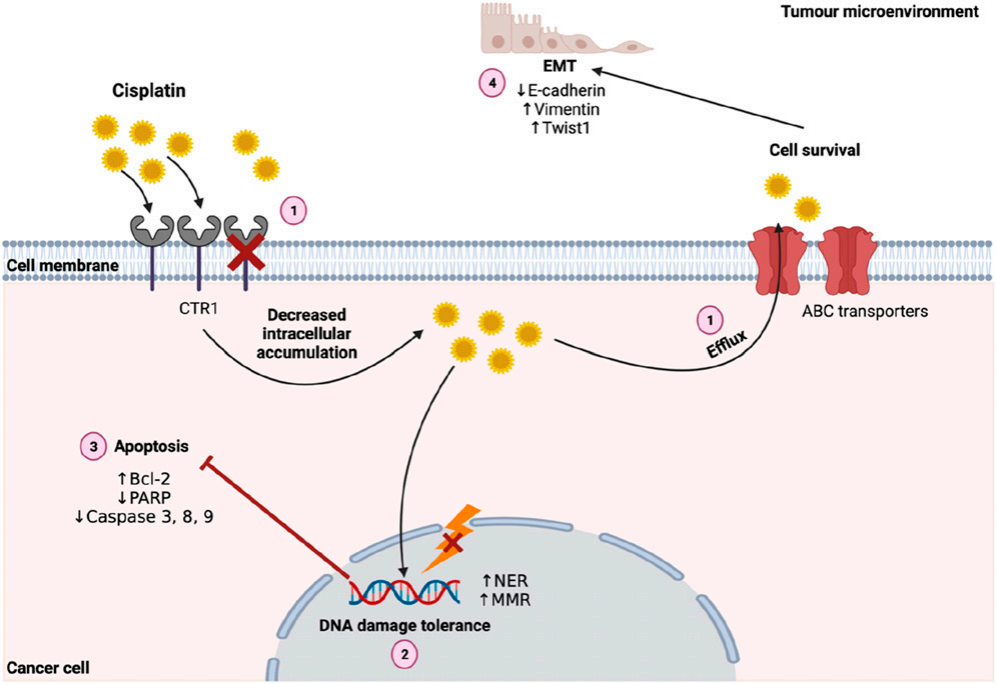
Overview of cisplatin treatment resistance. (1) Decreased intracellular accumulation of platinum compounds; (2) Increased DNA damage repair; (3) Inactivation of apoptosis; (4) Epithelial-mesenchymal transition. Abbreviations: CTR1: Copper transporter 1; EMT: Epithelial-mesenchymal transition; MMR: Mismatch repair; NER: Nucleotide excision repair; PARP: Poly (ADP-ribose) polymerase. Created with BioRender.com.

The chemotherapeutic agent cisplatin is a small-molecule platinum compound that acts as an alkylating anti-neoplastic agent used in several cancers.^12,13^ However, cisplatin possesses serious dose-limiting side effects such as nausea and vomiting, neuropathy, kidney damage, bone marrow suppression, hypomagnesemia, and sensorineural hearing loss.^14^ These dose-limiting effects can negatively affect clinical efficacy. The molecular mechanisms underlying cisplatin resistance (CPR) are usually complex and associated with, but not limited to the following features: (1) decreased intracellular accumulation of platinum compounds; (2) increased DNA damage repair; (3) inactivation of apoptosis; and (4) activation of epithelial-mesenchymal transition (EMT). This is extensively reviewed elsewhere.^11^ Figure 1 schematically depicts these processes.

Another approach which has also been used to investigate CPR, is a systems biological approach where gene expression analysis on HeLa cells showed that several genes are consistently upregulated or downregulated in CPR cells.^15^

## Genes that Promote Cervical Cancer Development

Genetic imbalance, i.e., predominance of pro-oncogenic genes or anti-tumor suppressor genes due to pre-existing genetics and/or acquired events can advance cervical cancer development by promoting treatment resistance.^16^ Genes commonly mutated in several cancers that are associated with treatment resistance, and their relevant anti-cancer agents are summarized in Table 1. However, studies that focus on gene alterations in the context of cervical cancer progression and drug resistance remain limited. These findings could therefore serve as a promising avenue to improve treatment resistance in cervical cancer patients.

**Table 1.** Genes Commonly Mutated in Several Cancers that are Associated with Treatment Resistance and Their Relevant Anti-Cancer Agents.

Mutation	Cancer	Treatment Resistance	Mechanism	Reference
*PIK3CA*	Breast cancer	Adriamycin	Decreased apoptosis	^17^
			Hyperactivation of PI3K/AKT pathway	
	Colorectal cancer (CRC)	FOLFOX (5-FU and oxaliplatin)	Hyperactivation of PI3K/AKT pathway	^18^
			Increase LGR5^+^ CRC stem cells	
	Her2^+^ breast cancer	Trastuzumab	Hyperactivation of PI3K/AKT pathway	^19^
	Cervical cancer	Cisplatin	Decreased MAPK activity	^20^
			Upregulate p21-activated kinases (PAKs)	
*TP53*	CRC	5-Fluorouracil (5-FU)	Decreased apoptosis	^21^
			Decreased PUMA and p21	
	Non-small cell lung cancer (NSCLC)	Cisplatin	Increased NRF2	^22^
	Lung cancer	Adriamycin	Decreased apoptosis	^23^
	Ovarian cancer	Taxane and platinum based adjuvant therapy	Decreased BAK	^24^
			Decreased p21	
			Increased mTOR	
	Cervical cancer	Cisplatin	Decreased apoptosis (loss of p53)	^12^
			DNA damage tolerance	
	NSCLC	Cisplatin	Persistent mTOR signalling	^25^
*KRAS*	NSCLC	Platinum-based chemotherapies	-	^26^
	Testicular cancer	Cisplatin	Increased NRF2	^27^
			Upregulated PI3K-AKT-mTOR pathway	^28^
*PTEN*	NSCLC	Radiotherapy	Increased AKT phosphorylation	^29^
	Endometrial cancer	Docetaxel	Decreased cyclin D1	^30^
	NSCLC	Cisplatin	-	^31^
		Gemcitabine		

### TP53

Mutations in the *TP53* gene are among the most common genetic alterations in several human malignancies.^32^ Somatic *TP53* mutations occur in several types of cancer at rates from approximately 38-50% in ovarian, esophageal, colorectal, head and neck and lung cancers to about 5% in primary leukemia, sarcoma, testicular cancer, malignant melanoma, and cervical cancer.^33^ Alterations in the *TP53* gene cause functional loss of p53, a tumour suppressor protein. During high-risk HPV integration, the E6 oncoprotein can bind to and control the function of p53. E6 interacts with E6-associated protein (E6-AP), a 100-kDa cellular protein that serves as a ubiquitin-protein ligase. E6-AP catalyzes multi-ubiquitination which results in the breakdown of p53 once it binds to the dimeric complex. It has been reported that with high-risk HPV integration, E6 is the only oncoprotein capable of triggering p53 breakdown (Tommasino et al, 2003). Furthermore, Li-fraumeni syndrome (LFS) is an extremely rare autosomal-dominant hereditary disorder characterized by a germline mutation in the tumour-suppression gene *TP53*, with an estimated 50-fold risk over the general population of developing several types of cancer.^34^ Although endometrial and ovarian cancers have been found to occur excessively in some families who have met criteria for LFS, their link to the syndrome is not definitely established.

In primary cervical tumours, which are HPV-negative, p53 mutations are very rare.^35^ Because the viral oncoprotein E6 binds to and inhibits the function of p53 protein, inhibition by HPV may be one cause of chemoresistance in cervical cancer.^36^ Mutated *TP53* can also regulate the expression of chemo- and radioresistant genes, one of which includes *MDR1*. *MDR1* mediates the resistance of tumour cells to various hydrophobic cytotoxic drugs.^37^ Furthermore, a study from India (LMIC) reported that the downregulation of *TP53* and its dysfunction in neoplastic tissue was associated with cervical cancer progression, where *p53* mRNA expression was downregulated in lower stages (stage IIA and IIB) compared to higher stages of cervical cancer.^38^ In another study, 3 out of 4 patients with *TP53* mutation showed highly aggressive tumour recurrence after concurrent chemoradiation (CCRT) within 10 months.^39^ Interestingly, it has also been shown that *TP53* mutations are observed more frequently in adenocarcinoma compared to squamous cell carcinoma (SCC).^40^ However, whether *TP53* gene mutations have an impact on prognosis and response to molecularly targeted therapies, in cervical cancer histotypes, requires further investigation. Promisingly, a clinical trial has compared the use of p53 expressing adenoviral vector in cervical cancer patients and found that when used in combination as neoadjuvant chemotherapy, tumour regression almost comparable to that in a cisplatin, vinblastine, and bleomycin (PVB) treatment group was observed .^41^

### KRAS (Kirsten Rat Sarcoma Viral Oncogene Homolog)

KRAS is an oncogene that encodes a small GTPase transductor protein called K-RAS, which is the most frequently mutated isoform in RAS-driven cancers (86%), followed by N-RAS (11%) and H-RAS (3%). Almost 98% of oncogenic *RAS* mutations are found on the active site amino acid residues G12, G13 and Q61, whose mutations impair intrinsic and GAP-mediated GTP hydrolysis. *KRAS* functions as a GTPase and plays an important role in regulating cell differentiation, proliferation, and survival,^42^ where mutations in *KRAS* can be detected in approximately 30% of all human cancers.^43^ Point mutations at these active site residues can lead to the accumulation of cellular GTP-bound RAS, which activates downstream signaling pathways. *KRAS* mutations constitute a poor prognostic marker in NSCLC and colorectal cancer, however, its prognostic characteristic in cervical cancer remains to be elucidated.^44,45^ To date, RAS proteins have not yielded successful targeted therapies. Although drugs have been designed to block pathways downstream of RAS, including RAF, MAPK-MEK and ERK,^46^ the efficacy thereof remains inconclusive. In one study, *KRAS* mutations were predominant in SCC of the cervix and were associated with HPV-18 infection. In patients with the *KRAS* mutation, distant metastasis and pelvic recurrence within the surgical or radiation area were documented in 29.6% and 11.1% of the patients, respectively. In these specific subtypes of cervical cancer, patients with a *KRAS* mutation had a worse prognosis.^47^ Additionally, Wright and co-authors indicated that out of 40 patients with adenocarcinoma, 8.8% presented with *KRAS* mutations which were not detected in patients with SCC.^48^ These *KRAS* mutations were typical G12 and G13 missense mutations within the guanine exchange factor domain. Previously, KRAS mutations have been classified as ‘undruggable’. Studies suggest that the presence of the KRAS G12 C allele is associated with worse survival than other *KRAS* mutations in patients with CRC cancer. In ongoing studies, Adagrasib (an inhibitor of the RAS GTPase family) demonstrated anti-tumour activity (CRC and NSCLC) in the KRYSTAL-1 phase I/II study conducted among heavily pretreated patients with KRAS G12 C mutations.^49,50^

### PIK3CA

*PIK3CA* is a gene that encodes the p110 alpha catalytic subunit of Class I PI3Kinase and can be amplified in copy number and activated by certain driving mutations. It has been shown that in cases of metastatic or recurrent cervical cancers, the PI3K/AKT/mTOR pathway is highly dysregulated.^51^ Furthermore, *PIK3CA* mutations are one of the most frequently detected mutations in cervical cancer and activating mutations in *PIK3CA* are associated with cancer cell survival, invasion, metastasis, angiogenesis, and apoptosis resistance.^52,53^ *PIK3CA* mutations are also associated with paclitaxel resistance.^54^ Many studies have reported that cervical cancer patients with *PI3KCA* mutations are associated with worse prognosis compared to those with wild-*type PI3KCA*, but this remains controversial.^55,56^ In a meta-analysis study, patients with the mutated *PIK3CA* genotypes had worse overall survival compared to patients with wild-type *PIK3CA,* primarily among those with SCC.^57^ In HeLa cells, the PI3K pathway is significantly activated in paclitaxel-resistant cells compared to parental cells. Combining paclitaxel with a PI3K-inhibitor induced apoptosis via Bax and PARP and enhanced drug sensitivity when compared to paclitaxel alone.^58^ Alpelisib, a PI3K inhibitor, was recently approved for the treatment of hormone receptor-positive (HR^+^) and human epidermal growth factor receptor 2 negative (HER2^−^) PIK3CA-mutant breast cancer. Although it is being investigated in several types of carcinomas, its effects in cervical cancer have not been established.^59^ In a recent study, Alpelisib suppressed *PIK3CA*-mutant cervical carcinoma cell proliferation and migration in vitro (ME-180) and in vivo.^60^ Furthermore, mutations in *PIK3CA* are also associated with higher rates of mutations in other genes of important cancer-associated pathways, such as the tyrosine kinase receptors/K-Ras/BRAF/MAPK and the Wnt/β catenin pathways.^61^

Another drug, Copanlisib (a PI3K inhibitor), is currently included in the phase II MATCH trial. This trial studies how well treatment specifically directed at genetic testing works in patients with solid tumours or lymphomas that have progressed after at least one line of standard treatment or for which no treatment approach exists.^62^

### PTEN (Phosphatase and Tensin Homolog)

One of the most fundamental characteristics of cancer cells is their ability to sustain proliferative signaling, especially via the phosphatidylinositol 3-kinase (PI3K) pathway. *PTEN* is mainly involved as a negative regulator in the PI3K/AKT/mTOR pathway, is a tumour suppressor gene, and regulates many cellular functions such as proliferation, survival, and genomic stability.^63^ *PTEN* contains two key domains for its tumour suppressor functions, namely, the phosphatase domain and the C2 domain. Unsurprisingly, deregulation of *PTEN* has been observed in many human cancers^64^ such as cervical, ovarian, endometrial, prostate and breast cancers, where 13% of overall cases presented with mutated *PTEN*.^65^ According to the Catalogue of Somatic Mutations in Cancer (COSMIC) data set, approximately 30% of *PTEN* alterations in cervical cancer patients are related to gene under expression.^66^ Loures and co-authors demonstrated that *PTEN* expression intensity was lower in SCC patients compared to control patients (benign cervix) and was not associated with tumour expression of p53.^67^

**Figure 2.**
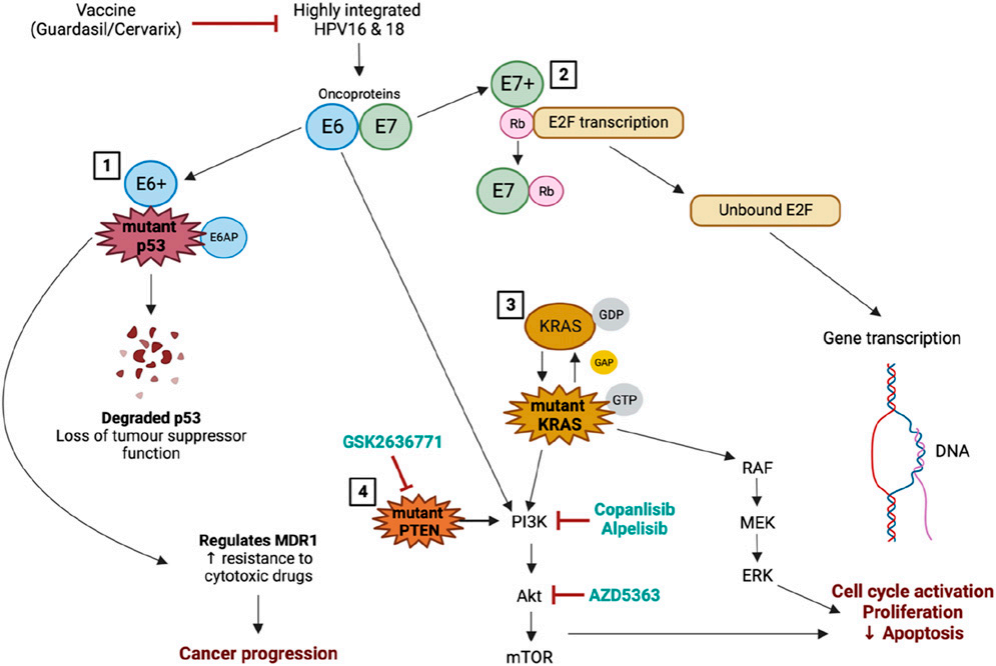
Genetic mutations contribute to cervical cancer progression. Several mutations (*de novo* or acquired) contribute to treatment resistance by preventing cancer cell death and instead promotes cancer progression. Currently, several inhibitors of these genetic mutations are being investigated in phase I and phase II clinical trials which could promote more favourable treatment responses in cancer patients. (1) During high-risk HPV integration, E6 can bind to and control the function of p53. E6 interacts with E6-associated protein (E6AP), which catalyzes multi-ubiquitination resulting in the breakdown of p53 once it binds to the dimeric complex. Mutated *TP53* can also regulate the expression of chemo- and radioresistant genes, including *MDR1*. *MDR1* mediates the resistance of tumour cells to various hydrophobic cytotoxic drugs. (2) E7 disrupts the interaction between Rb and E2F, resulting in the release of E2F in its transcriptionally active form. This E7-mediated conversion of E2F to its activator forms stimulates cell cycle activation and proliferation. (3) Transition from KRAS-GDP to KRAS-GTP (mutant KRAS) requires GAPs proteins. Downstream signalling of KRAS induces cellular proliferation. (4) *PTEN* is mainly involved as a negative regulator in the PI3K/AKT/mTOR pathway, however, once dysregulated, it promotes cell cycle activation/proliferation. Abbreviations: E2F: E2 promotor binding factor; ERK: Extracellular signal-regulated kinase; GAP: GTPase activating proteins; GDP: Guanosine diphosphate; GTP: Guanosine triphosphate; HPV: Human papilloma virus; KRAS: Kirsten rat sarcoma viral oncogene homolog; MEK: Mitogen-activated protein kinase; P: Phosphorylate; PTEN: Phosphatase and tensin homolog; RAF: Rapidly accelerated fibrosarcoma; Rb: Retinoblastoma. Created with Biorendor.com.

Currently, *PTEN* mutation is an inclusion criterion in a phase 1 clinical trial including cervical cancer patients (NCT01226316, active not recruiting). In this study, the safety and tolerability of AZD5363, an inhibitor of AKT, is being investigated in patients with advanced cancer. Additionally, this mutation is also an inclusion criterion in a sub study of a phase 2 clinical trial (NCT02465060).^62^ In this study, patients receive PI3K-beta inhibitor, GSK2636771. Figure 2 summarizes the effects of genetic mutations on cervical cancer progression.

Therefore, the molecular characterization of cervical tumours provides clinicians with the opportunity to qualitatively assess tumour burden and possibly predict clinical behaviour in individual patients. Metastatic lesions are not always accessible by needle aspirations, which is a hurdle for getting personalized information of potential targets for therapy or resistance mechanisms.^68^ Characterizing tumours by means of a biopsy could enable more effective treatment choices and potentially decrease the incidence of unnecessary toxicity and side effects in patients receiving traditional chemotherapy and targeted therapy.^69^ Taken together, the study of molecular characterization is enticing and has the potential to become an essential component of cervical cancer management.

## Appendix

### Abbreviations

CPR: Cisplatin resistance
HPV: Human papillomavirus
KRAS: Kirsten rat sarcoma viral oncogene homolog
PIK3CA: Class I phosphatidylinositol 3-kinase
PTEN: Phosphatase and tensin homolog
SCC: Squamous cell carcinoma.
